# Amyloid peptides ABri and ADan show differential neurotoxicity in transgenic *Drosophila* models of familial British and Danish dementia

**DOI:** 10.1186/1750-1326-9-5

**Published:** 2014-01-09

**Authors:** María S Marcora, Agata C Fernández-Gamba, Luz A Avendaño, Cecilia Rotondaro, Osvaldo L Podhajcer, Rubén Vidal, Laura Morelli, María F Ceriani, Eduardo M Castaño

**Affiliations:** 1 From Fundación Instituto Leloir, Av. Patricias Argentinas 435, Buenos Aires C1405BWE, Argentina; 2 Instituto de Investigaciones Bioquímicas de Buenos Aires, Consejo Nacional de Investigaciones Científicas y Técnicas (CONICET), Av. Patricias Argentinas 435, Buenos Aires C1405BWE, Argentina; 3 Indiana Alzheimer Disease Center and Department of Pathology and Laboratory Medicine, Indiana University School of Medicine, 635 Barnhill Dr, MSB A136, Indianapolis, IN 46202, USA

**Keywords:** Alzheimer’s disease, Familial British dementia, Familial Danish dementia, ABri, ADan, Neurotoxicity, *Drosophila*

## Abstract

**Background:**

Familial British and Familial Danish dementias (FBD and FDD, respectively) are associated with mutations in the BRI_2_ gene. Processing of the mutated BRI_2_ protein leads to the accumulation in the brain of the 34-mer amyloid Bri (ABri) and amyloid Dan (ADan) peptides, accompanied by neurofibrillary tangles. Recently, transgenic mice successfully reproduced different aspects of FDD, while modeling of FBD *in vivo* has been more difficult. In this work we have modeled FBD and FDD in *Drosophila* and tested the hypothesis that ABri and ADan are differentially neurotoxic.

**Results:**

By using site-directed insertion, we generated transgenic lines carrying ABri, ADan, Bri_2_-23 (the normal product of wild-type BRI_2_ processing) and amyloid-β (Aβ) 1–42 as a well-characterized neurotoxic peptide, alone or with a His-tag. Therefore, we avoided random insertion effects and were able to compare levels of accumulation accurately. Peptides were expressed with the *GAL4*-Upstream Activating Sequence (*UAS*) system using specific drivers. Despite low levels of expression, toxicity in the eye was characterized by mild disorganization of ommatidia and amyloid peptides accumulation. The highest toxicity was seen for ADan, followed by Aβ42 and ABri. Pan-neuronal expression in the CNS revealed an age-dependent toxicity of amyloid peptides as determined by the ability of flies to climb in a geotaxis paradigm when compared to Bri_2_-23. This effect was stronger for ADan, detected at 7 days post-eclosion, and followed by ABri and Aβ42, whose toxicity became evident after 15 and 21 days, respectively. Histological analysis showed mild vacuolization and thioflavine-S-negative deposits of amyloid peptides. In contrast, the over-expression of amyloid peptides in the specific subset of lateral neurons that control circadian locomotor activity showed no toxicity.

**Conclusions:**

Our results support the differential neurotoxicity of ADan and ABri in the *Drosophila* eye and CNS at low expression levels. Such differences may be partially attributed to rates of aggregation and accumulation. In the CNS, both peptides appear to be more neurotoxic than wild-type Aβ42. These *Drosophila* models will allow a systematic and unambiguous comparison of differences and similarities in the mechanisms of toxicity of diverse amyloid peptides associated with dementia.

## Background

Familial British Dementia (FBD) and Familial Danish Dementia (FDD) are autosomal dominant neurodegenerative disorders associated with mutations in the BRI_2_ gene on chromosome 13 (also named ITM2B) [1,2]. FBD usually starts in the fifth decade of life with progressive dementia, spasticity and ataxia leading to death in ~9 years [3]. FDD starts earlier, before 30 years of age with cataracts, visual loss followed by impaired hearing, progressive cerebellar ataxia and late dementia. Patients die within the sixth-seventh decade of life [4]. A prominent neuropathological finding in FBD and FDD patients is the accumulation of amyloid proteins in the walls of small arteries with a widespread distribution. This includes the cerebral cortex, leptomeninges, cerebellum, brain stem and white matter [5,6]. In addition, parenchymal amyloid deposits and neurofibrillary tangles are consistently found with a notorious and severe compromise of the hippocampus. In this regard, FBD and FDD (together with some cases of hereditary prionoses) are closely similar to Alzheimer’s disease (AD), the major cause of dementia in aging populations. A further similarity between FBD, FDD and AD is that amyloid deposits are made of short peptides generated in the brain by internal proteolysis of larger transmembrane precursor proteins. These peptides of ~4 kDa, are: amyloid β (Aβ) in AD [7,8], ABri in FBD and ADan in FDD [1,2]. In FBD, a missense mutation at the BRI_2_ stop codon leads to the generation of the ABri peptide sequence [1]. In FDD, a 10-nucleotide duplication insertion causes a frame shift and the generation of the ADan sequence [2]. Both 34-residue peptides (ABri and ADan) and the normal peptide product of wild-type BRI_2_ (Bri_2_-23) are released by furin and other subtilisin/kexin-like proprotein convertases (PCs) by cleavage of the BRI_2_ carboxyl-terminus along the secretory pathway [9].

**Figure 1:**
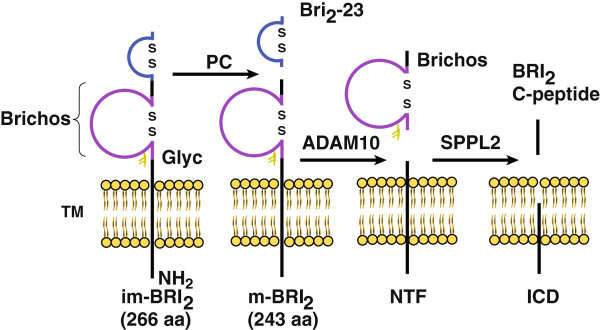
**Proteolytic processing of BRI**_**2**_**.** Schematic diagram representing the proteolytic events of BRI_2_ processing. Proprotein convertases (PC) release a C-terminal 23-residue peptide (Bri_2_-23). Cleavage by ADAM10 of the BRI_2_ ectodomain releases the Brichos domain to the extracellular space and leaves an N-terminal fragment (NTF) attached to the membrane. NTF is further processed by signal peptide peptidase-like 2 (SPPL2) generating an intracellular domain (ICD) and an extracellular BRI_2 –_C-terminal peptide (BRi_2-_C peptide). Instead of Bri_2_-23, cleavage of the mutant BRI_2_ by PC releases ABri in FBD or ADan in FDD, respectively (not depicted). im-BRI_2,_ immature BRI_2;_ m-BRI_2_, mature BRI_2_. Glyc, glycosylation; TM, trans-membrane; ICD, intracellular domain.

Processing of BRI_2_ seems to be complex, involving several proteases in addition to PCs. The pro-protein and mature BRI_2_ (m-BRI_2_) protein may also be cleaved by a disintegrin and metalloproteinase domain-containing protein 10 (ADAM10), releasing the Brichos domain [10] and an N-terminal fragment (NTF). The NTF is also the subject of additional proteolysis by signal peptide peptidase-like 2 (SPPL2), releasing an intracellular domain (ICD) and the BRI_2_ C-peptide (Figure 1) [11]. To date, the biological roles of BRI_2_ and the pro-peptide Bri_2_-23 have not been elucidated. *In vitro* studies showed that oligomers of ABri and ADan are toxic to neuronal cell lines [12,13].

Recently, transgenic mouse models for FDD have been generated. The first reported line carries a mutant BRI_2_ under the mouse prion protein promoter and after 6 months of age shows extensive vascular deposition, parenchymal ADan accumulation, gliosis and an increase of phosphorylated-tau immunoreactivity [14]. When this transgenic animal was crossed with tau-P301S transgenic mice (Tg-Tau P301S), there was an increase of tau accumulation, phosphorylation and caspase cleavage of tau at Asp421 [15]. A knock-in (KI) mouse, carrying the FDD mutation in endogenous BRI_2_ has also been generated although it did not show detectable brain abnormalities [16]. In addition, two other transgenic lines that overexpress BRI_2_ containing the FDD mutation have been produced [17]. The FDD-like line with higher expression displays ADan accumulation in the hippocampus and meningeal vessels after 2 months of age with a marked age-dependent increase in amyloid deposition, particularly in the microvasculature. In addition, when crossed with Tg-tau P301S mice, these animals show a significant increment in the accumulation of hyperphosphorylated tau as compared to the Tg-tau P301S alone. Therefore, mouse models of FDD that overexpress mutant BRI_2_ in the brain recapitulate several key features of the human disease.

Regarding FBD, the development of a transgenic animal model reproducing basic lesions of the disease has been more elusive. Lines of transgenic mice carrying the FBD mutation have been generated and despite high levels of mutant BRI_2_ expression, no brain pathology was detected. Moreover, ABri peptide did not accumulate in the brain and only minimal amounts were detected by immunoprecipitation even after exogenous furin overexpression [18]. A second approach, using the KI strategy, showed that mice carrying the FBD mutation in one mouse BRI_2_ allele developed a significant deficit in hippocampal-dependent memory tasks by the age of 9 months without neuropathology or ABri deposition [19]. However, a reduction in BRI_2_ was detected in synaptic vesicles as compared to wild-type mice. These results together with the finding of similar memory deficits in BRI_2_ haplo-deficient mice (Bri2^+/-^) and lower levels of BRI_2_ in FBD brains has led to the hypothesis that loss of function of BRI_2_ may be a contributing factor to the development of dementia in FBD patients [19]. Other studies have reported that BRI_2_ may modulate amyloid β precursor protein (AβPP) processing in transfected cells and in transgenic mice leading to a reduced Aβ production and aggregation [20,21].

*Drosophila melanogaster* has been extensively used to reproduce basic aspects of several neurodegenerative diseases including AD, Parkinson disease, frontotemporal dementia, polyglutamine diseases, non-coding trinucleotide repeat expansion disease, amyotrophic lateral sclerosis and prion diseases [22-25]. The first fly model for AD was designed to over-express human AβPP, human β-secretase and presenilin1 mutants associated with familial AD. These animals showed Aβ accumulation and shortened life span [26]. Other groups have generated transgenic flies targeting Aβ directly to the secretory pathway. These flies developed age-dependent amyloid accumulation and reduced life span accompanied by defects in locomotion, memory and learning [27-30]. Further studies of these lines showed that Aβ caused defects in axonal transport, synaptic integrity and mitochondrial mislocalization [31-33].

In this study we report the generation and characterization of transgenic flies that over-express ABri, ADan and the normal product of BRI_2_, Bri_2_-23, by site-directed insertion. For comparison, a line expressing Aβ1-42 was also generated. Although the toxicity of wild-type Aβ and Aβ mutants in flies has been extensively described [26-41], the strategy used in this study included Aβ as a reference for comparison with ABri and ADan, for which no *Drosophila* models have been reported. Our results support the neurotoxicity of both FBD and FDD-associated peptides in contrast to Bri_2_-23. In addition, important differences were appreciated in terms of degree of toxicity and vulnerability of neuronal types. Moreover, this is the first report of the toxic effects of ABri expression in an animal model.

## Results and discussion

### Generation of transgenic lines

**Figure 2:**
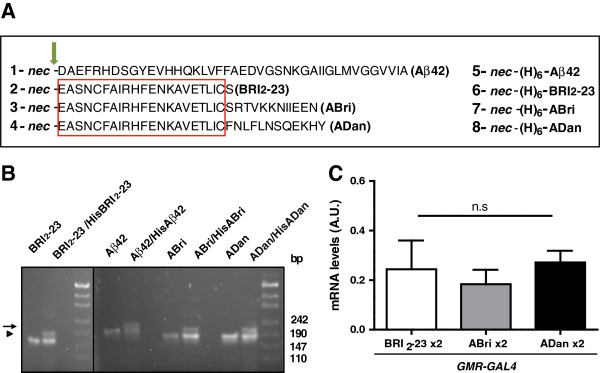
**Generation of transgenic lines with similar expression levels. A**, schematic representation of the fusion peptides used to generate transgenic flies. Amino-acid single-letter code is used. *Nec*, the signal peptide of necrotic (MASKVSILLLLTVHLLAAQTFAQ); (H)_6_ indicates the 6 × His-tag. The arrow indicates the site of cleavage in the secretory pathway. The rectangle encloses the sequence shared by Bri_2_-23, ABri and ADan. **B**, agarose gels showing the PCR products obtained from genomic DNA of transgenic lines carrying one copy of untagged (arrowheads) or two copies: one untagged and the other with a 6 × His-tagged peptide DNAs (arrows). On the right, molecular markers in base-pairs (bp). **C**, similar levels of mRNA expression of Bri_2_-23, ABri and ADan in the eye as determined by QRT-PCR normalized with tubulin mRNA. Bars represent the mean ± SEM of 3 independent experiments. A.U., arbitrary units.

To examine and compare the effects of ABri, ADan and Aβ42 over-expression in *Drosophila*, we generated transgenic lines carrying the sequence of each amyloid peptide fused to the signal peptide of *Drosophila Necrotic* (*Nec*) that targets the protein to the secretory pathway This strategy has been used successfully with Aβ [29] and circumvents possible difficulties due to an insufficient processing of BRI_2_ (particularly in the case of ABri, as suggested by the mouse models). To avoid positional effects associated with random insertion in the genome, we used the φ-recombinase-based system [42] to direct insertion of transgenes at a specific site on chromosome 3. Since there are no specific antibodies to Bri_2_-23, we generated a set of constructs with a 6 x His-tag at their amino-termini (Additional file 1). This allowed us to compare the levels of accumulation of each peptide by using the same antibody. All the peptides designed in this study are schematically shown in Figure 2A. PCRs with specific primers were used to assess the insertion of the correct cDNAs (Figure 2B). The “site-directed” strategy allows a better comparison of the effect caused by each peptide because the levels of mRNA expression should be similar. Accordingly, Quantitative Real-Time PCR (QRT-PCR) using the same set of primers showed no significant differences in the expression levels of Bri_2_-23, ABri and ADan mRNAs (Figure 2C).

### Expression and processing of Bri_2_-23, Aβ42, ABri and ADan

**Figure 3:**
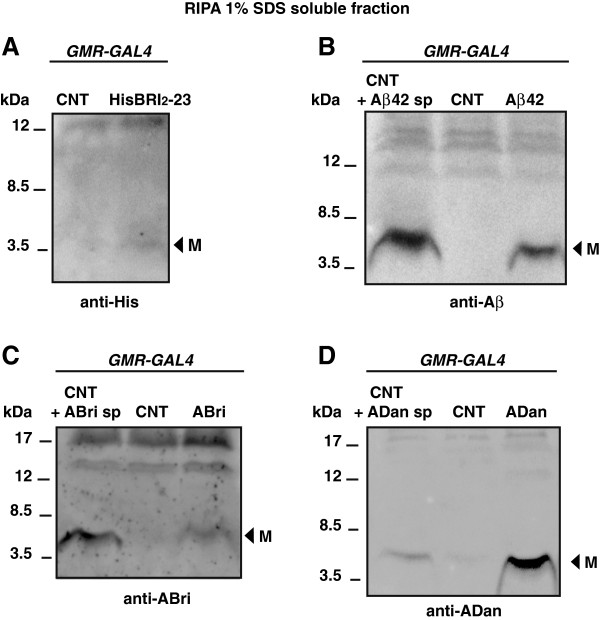
**Amyloid peptides are properly processed in *Drosophila *eye.** RIPA-soluble proteins extracted from flies expressing two copies of each peptide in the eye using the *GMR*-*GAL4* driver. **A**, immunoreactivity with anti-His showing a very faint 3 kDa band consistent with HisBri_2_-23 monomer (arrowhead). **B**, Western blot with 6E10 showing the 4.5 kDa monomeric Aβ42 in transgenic line (arrowhead). Synthetic (sp) Aβ42 was added to wild type *Drosophila* protein extract to compare molecular masses. **C**, immunoreactivity using anti-ABri revealed a specific band with the expected sizes of a monomer (arrowhead); on the right, synthetic (sp) ABri peptide added to the protein extract from a non-transgenic fly. **D**, Western blot using anti-ADan show the presence of a band (arrowhead) consistent with ADan monomer in the transgenic line. Wild type *Drosophila* protein extract was spiked with sp ADan peptide for comparison. M, monomers. CNT, control flies (*GMR-GAL4*/+). In all lanes, RIPA-soluble proteins from 15 head homogenates (150 μg) were loaded. Representative gels from more than 3 independent experiments for each genotype are shown.

To examine whether the fusion peptides were correctly targeted and cleaved to release two copies of Bri_2_-23, Aβ42, ABri and ADan, peptides were expressed in the eye using the Glass Multiple Reporter (*GMR-GAL4*) driver and the *GAL4-UAS* bipartite system. Ten days post-eclosion (p.e), homogenates from transgenic and control fly heads were analyzed by Western blots. In the *GMR-GAL4*/HisBri_2_-23 line, anti-His showed a very faint band of ~3 kDa (at the limit of detection) compatible with a properly processed Bri_2_-23 (Figure 3A). The Aβ42 monomer was clearly detected with monoclonal 6E10 antibody (Figure 3B). Regarding ABri, a specific band with the size of a monomer was detected with anti-ABri antibody (Figure 3C). The processing of ADan was assessed with a specific antibody directed to the peptide carboxyl-terminus, which showed a 4 kDa band consistent with ADan monomer (Figure 3D). It is of note that differently from other reports using Aβ-transgenic *Drosophila*, mouse models of ADan or brain tissue from FBD and FDD patients, no SDS-resistant high order oligomeric species were detected in our transgenic lines (Additional file 2). In addition, the bulk of amyloid peptides were solubilized in buffer containing 1% Triton X-100 and 1% SDS, while only traces of Aβ42 and ADan remained as a formic-soluble fraction (Additional file 2). These results suggest that the proteins are correctly processed along the secretory pathway, generating mostly soluble peptides with small amounts of insoluble aggregates.

### Toxicity of Aβ42, ABri and ADan in the eye

**Figure 4:**
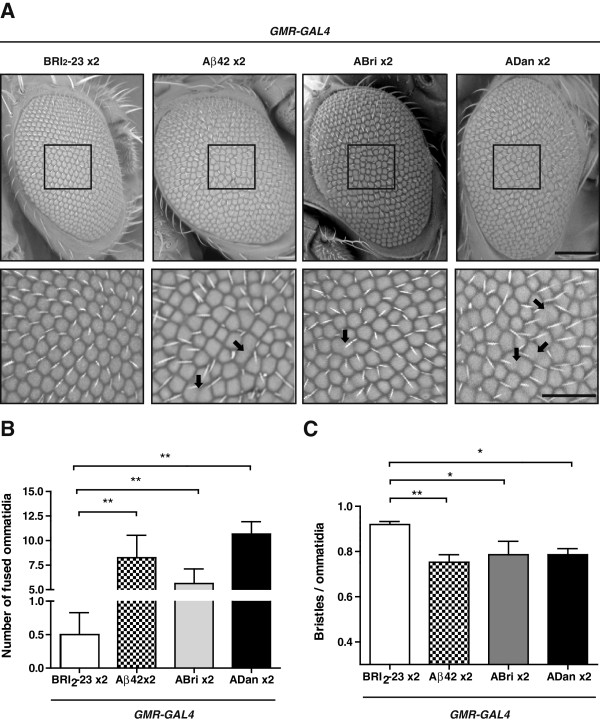
**Differential toxicity of Aβ42, ABri and ADan in the eye. A**, morphological analysis of eye integrity from flies expressing Bri_2_-23, Aβ42, ABri and ADan. Top panel, ESEM microphotographs indicating the selected area (135 × 135 μm) used to count the number of ommatidia and bristles.The area used for quantification of fused ommatidia was slightly larger (195 × 170 μm) and it is not shown for clarity. Bottom panel, higher magnification of the selected regions as delineated in the top panel. Fused ommatidia are indicated with arrows. Bar scales, 100 μm and 50 μm for top and bottom panels, respectively. **B**, quantification of fused ommatidia in the selected area for each genotype. **C**, quantification of bristles in the selected area for each genotype. One- way ANOVA followed by Dunnett’s comparison test analysis was performed. Eight eyes per genotype, randomly taken from 5 independent biological experiments were analyzed. Data are expressed as mean ± SEM. Asterisks indicate statistically significant differences (*p < 0.05, **p < 0.01).

At 10 days p.e, eyes were morphologically examined for toxicity by light microscopy and scanning electron microscopy (ESEM). When one or two copies of Aβ42, ABri and ADan were expressed in the eye using *GMR-GAL4* and flies maintained at 25°C, no toxic effects were detected (not shown). By raising temperature to 28°C, an expected mild toxicity was seen in heterozygous *GMR-GAL4*/+ lines, as reported [43]. However, such effect was greatly reduced in the *GMR-GAL4*/Bri_2_-23 line, likely due to titration of free *GAL4* to the *UAS* repeats available for *GAL4* binding (Additional file 3). Therefore, we used *GMR-GAL4*/Bri_2_-23 as a control to compare the effects of Aβ42, ABri and ADan expression. The eyes from *GMR-GAL4*/Bri_2_-23 flies showed few scattered ommatidia of different sizes, but this defect was much lower than the disorganization seen in the amyloid-expressing lines. Light microscopy (not shown) and ESEM micrographs revealed a pattern of ommatidia disarray in the amyloid- expressing eyes related to BRI_2_-23 (Figure 4A). The penetrance of these phenotypes was 90-100% in all cases. Since the phenotype was mild, the extent of toxicity was assessed by counting the number of ommatidia, ommatidia fusions and number of bristles in a representative central area of the eye for each genotype. The number of fused ommatidia was significantly higher for ADan, Aβ42 and ABri as compared to BRI2-23 (Figure 4B) while the number of bristles per ommatidia was significantly lower in flies expressing the amyloid peptides as compared to control (Figure 4C). In addition, there was a significant loss of ommatidia in Aβ42 and ADan-expressing lines as compared to BRI_2_-23 (Additional file 3). Overall, the degree of toxicity reflected as ommatidia size heterogeneity and fusion tended to be higher for ADan and Aβ42 relative to ABri. These phenotypes obtained with Aβ42, ABri and ADan in our system were subtle as compared to the typical full-rough eye as reported for flies expressing high levels of Aβ [37]. When we compared our Aβ42 line with the reference line Aβ42 Hj2.12, the latter presented ~2.5-fold higher Aβ42 accumulation as measured by ELISA (7.5 vs 17.5 ng/mg total protein, respectively) and a more severe eye toxicity (Additional file 3), indicating that the “site-directed lines” used here have relatively low levels of expression, and yet, a toxic effect in the eye can be detected.

**Figure 5:**
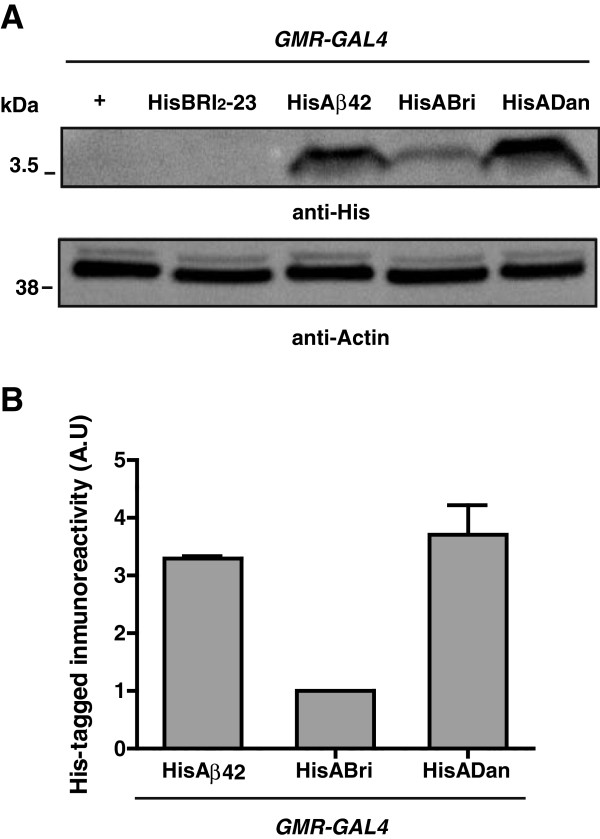
**Analysis of His-tagged amyloid protein levels. A**, Western blots of fly head homogenates expressing two copies of His-tagged peptides in the eye. Top panel, anti-His shows different levels of monomeric HisAβ42, HisABri and HisADan accumulation in transgenic flies after 10 days p.e. In this experiment, the expression levels of HisBri_2_-23 were below the limit of detection. Bottom panel, Western blot of the same membrane re-probed with anti-actin antibody for loading normalization. **B**, quantification of HisAβ42 and HisADan levels relative to HisABri. At least 3 independent experiments were performed. A.U., arbitrary units.

In order to examine the accumulation levels of amyloid peptides *in vivo* in a comparable way, two copies of the His-tagged constructs were expressed in the eye and assessed by Western blots using anti-His. As shown in Figure 5, the reactivity of HisADan and HisAβ42 were ~4 fold and ~3 fold higher than HisABri, respectively.

To evaluate the possible effect of the His tag upon amyloid peptides, transgenic lines carrying two copies of tagged or untagged peptides were analyzed by Western blots using amyloid-specific antibodies (Additional file 4). In the case of HisAβ42, there was a ~3 fold increase as compared to Aβ42 while there were no differences between ADan and HisADan. The effect upon ABri could not be accurately measured due to the very low signal and high background of the blots. These results raise the possibility that, at least for ADan (for which the His tag had no effect) its levels of accumulation may correlate with toxicity, as suggested for Aβ42. Further *in vivo* and *in vitro* studies may help to clarify the mechanisms by which the His tag imposes differential effects on amyloid peptides accumulation.

### Aβ42, ABri and ADan show different patterns of non-fibrillar deposition in the eye

**Figure 6:**
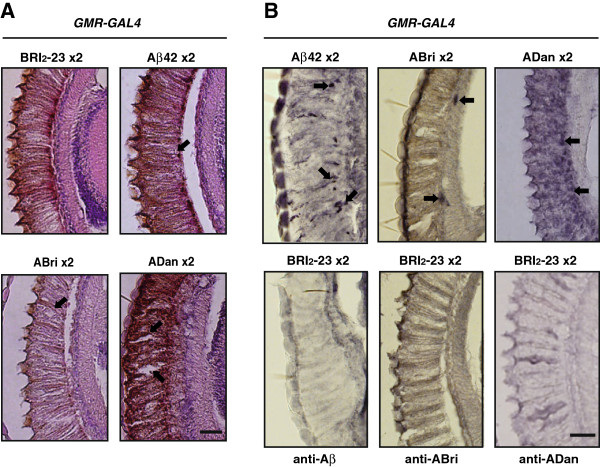
**Accumulation of amyloid peptides in the retina. A**, paraffin sections of the retina stained with H&E showing disorganization of ommatidia (arrows). Scale bar = 25 μm. **B**, horizontal sections from the retina of transgenic lines: *GMR-GAL4*/Aβ42 × 2, *GMR-GAL4*/ABri × 2 and*GMR-GAL4*/ADan x 2 were immunostained with 6E10, anti-ABri and anti-ADan, respectively and sections from *GMR-GAL4*/Bri_2_-23 x 2 control lines probed with the amyloid peptide-specific antibodies. Arrows indicate immunoreactivity consistent with amyloid peptide deposits. Images represent the results of at least 2 independent biological experiments. Scale bar = 20 μm.

Histological analysis of retina paraffin sections stained with hematoxilin and eosin (H&E) were consistent with the eye external phenotype, showing mild disorganization and heterogeneity in the sizes of ommatidia in *GMR*-*GAL4*/Aβ42, *GMR*-*GAL4*/ABri and *GMR-GAL4*/ADan as compared to *GMR*-*GAL4*/ Bri_2_-23 (Figure 6A).

The accumulation of Aβ42, ABri and ADan in the eye (expressing two copies of each peptide under *GMR-GAL4*) was studied by immunohistochemistry with specific antibodies on paraffin sections of the fly heads (Figure 6B). Specificity of staining was assessed by using 6E10, anti-ABri and anti-ADan antibodies upon eye sections of *GMR-GAL4*/Bri_2_-23 lines and unrelated IgG as primary antibodies. Aβ42 staining showed deposits in the retina following rhabdomers topology that were more abundant toward the basal ommatidial region. ABri immunoreactivity was scattered, showing discrete deposits in ommatidia close to the lamina different from ADan distribution that was mainly along the inner ommatidial rhabdomers with a stronger immunoreactivity. Thioflavine-S (ThS) staining of similar sections was negative for all peptides (not shown). These findings suggest that in the *Drosophila* eye, and despite similar levels of mRNA transcription, non-fibrillar Aβ42, ABri and ADan induced different degrees of toxicity, possibly related to different levels of accumulation and distribution.

### Toxicity of Aβ42, ABri and ADan in the CNS

**Figure 7:**
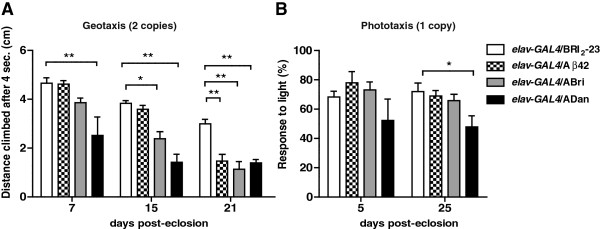
**Age and amyloid peptide-dependent toxicity in the CNS.** Pan-neuronal transgene expression was induced using the *elav-GAL4* driver. **A**, negative geotaxis assays were performed at the indicated times with flies expressing two copies of the transgenes. The slight decline in climbing performance with age in non-transgenic flies has been reported [reviewed in 36]. ADan expression was associated with reduced climbing at 7 days of age while the toxic effect of ABri and Aβ42 was seen at 15 and 21 days, respectively. Climbing was expressed in cm as the mean ± SEM. Data from 5 technical replicates from each of 3 independent biological experiments per genotype were analyzed. **B**, phototaxis assays were performed at the indicated times with flies expressing one copy of each transgene. At 25 days p.e, flies expressing ADan had a lower light response in comparison to Bri_2_-23, Aβ42 or ABri. Results from 2 technical replicates from each of 3 independent biological experiments per genotype are shown. Phototaxis was expressed as the percentage (mean ± SEM) of flies that responded to light. Asterisks indicate statistically significant differences, (*p < 0.05; ** p < 0.01; *** p < 0.001). Geotaxis and Phototaxis data were statistically analyzed by two-way repeated measures ANOVA, followed by Bonferroni’s comparison test for all genotypes and time points which are shown in Additional file 5.

To study Aβ42, ABri and ADan neurotoxicity in the CNS, peptides were expressed with the pan-neuronal driver *elav-GAL4*. Lines expressing Bri_2_-23 were used as negative controls and two behavioral paradigms were tested; negative geotaxis (to evaluate locomotor coordination) and positive phototaxis (to evaluate response to light). In the geotaxis assay, flies expressing one copy of each transgene and kept at 25°C showed no climbing defects (not shown). However, flies expressing two copies of amyloid peptides and kept at 28°C after eclosion showed behavioral defects that worsened with age (Figure 7A). After 7 days p.e, ADan-expressing flies displayed a significant impairment in climbing ability (in cm) compared to Bri_2_-23 (2.5 ± 0.7 vs 4.6 ± 0.2, respectively, p < 0.001) while in Aβ42 (4.6 ± 0.2) and ABri (3.8 ± 0.2) there was no detectable toxicity. After 15 days, flies expressing ABri showed a significant toxicity compared to Bri_2_-23 (2.3 ± 0.3 vs 3.8 ± 0.1 respectively, p < 0.01), while Aβ42 effect was only evident after 21 days as compared to Bri_2_-23 (1.5 ± 0.3 vs 3 ± 0.2, respectively, p < 0.01). Therefore, the three amyloid peptides were capable of causing a significant worsening of climbing ability in the geotaxis assay. However, Aβ42 and ABri neurotoxicity was rather mild and strongly age-dependent, as reported for other Aβ *Drosophila* models [32,33]. ADan, in turn, was highly toxic at the first time-point analyzed. In the phototaxis assay, flies expressing one copy of each peptide and kept at 25°C showed that only ADan was capable of exerting a significant impairment in 25 days-old flies (p < 0.05) reinforcing the higher toxicity of this peptide (Figure 7B and Additional file 5). These results were unlikely due to locomotor defects since flies expressing one copy of amyloid peptides under the same conditions showed no deficits in climbing assays, as mentioned above. Next, a specific group of neurons was tested for their vulnerability to amyloid peptides. The small and large lateral ventral neurons is a key subgroup of neurons that produce the neuropeptide pigment dispersing factor (*pdf*) and control circadian locomotor activity. We used the *pdf*-*GAL4* driver to direct expression of two copies of Bri_2_-23, ABri, ADan and Aβ42 to these cells. Neurodegeneration of *pdf*-neurons results in a dysregulation of the circadian rhythm with aging [44]. The analysis of circadian locomotor activity in flies expressing amyloid peptides showed normal rhythm and period both in young (5-days old) and aged (21-days old) individuals as compared to *pdf*-*GAL4*/ Bri_2_-23 or *pdf*-*GAL4*/+ (Additional file 6). This result was interesting although not unexpected, according to a previous report in which no circadian behavioral phenotype was observed in *pdf* neurons expressing the Arctic Aβ mutant [45]. Taken together, our behavioral experiments point at a different vulnerability of neuronal populations to amyloid peptides toxicity.

### Histological analysis and immunostaining in the CNS

**Figure 8:**
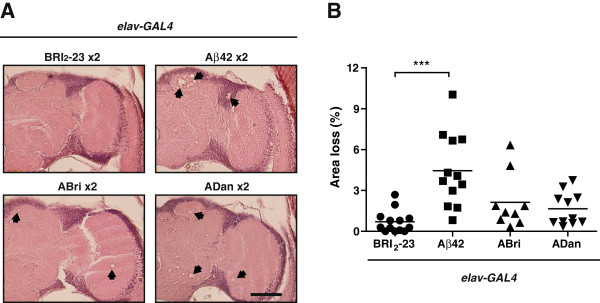
**Histological analysis of fly brains. A**, frontal sections of 21-day-old flies were stained with H&E and examined by bright-field microscopy. Vacuolization (>3 μm) of brain tissue was significantly higher in lines expressing Aβ42 as compared to Bri_2_-23, ABri and ADan. **B**, percentage of the area of tissue loss expressed as mean ± SEM. All lines expressed 2 copies of each peptide and 8–10 hemispheres per genotype were used for quantification. Asterisks indicate significant differences (One-way ANOVA, followed by Dunnett’s multiple comparison test). Scale bar = 50 μm.

**Figure 9:**
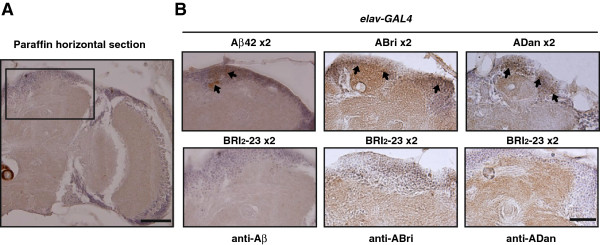
**Specific accumulation of amyloid peptides in the *Drosophila *CNS. A**, low magnification micrograph of brain section indicating the selected area from which immunohistochemistry is shown. **B**, amyloid deposits in the CNS were assessed with specific anti-amyloid peptide antibodies in lines expressing 2 copies of each transgene. Microphotographs of frontal paraffin sections showed discrete aggregates of Aβ42 and more widespread ABri and ADan deposits which were detected mainly in the supraesophagical brain, both in neurons and neuropil (arrows). No staining was detected in Bri_2_-23 line, used as a negative control for antibody specificity. Representative images from 3 independent biological experiments are shown. Scale bar = 50 μm.

To assess the effect of amyloid accumulation on the integrity of the CNS, two copies of each peptide were expressed under the pan-neuronal driver *elav-GAL4* and brain paraffin sections stained with H&E were examined (Figure 8A) under light microscopy. Specifically, we looked at the degree of vacuolization, a widely used parameter of neuronal death [46,47]. Amyloid peptides expression increased vacuolization, quantified as the loss of tissue area in cortical neurons and neuropil that was significantly higher for Aβ42 as compared to Bri_2_-23 (p < 0.001) while ABri and ADan showed a non-significant tendency (Figure 8B). Next, the accumulation of amyloid peptides in the brain was assessed by immunohistochemistry with specific antibodies using *elav*-*GAL4*/Bri_2_-23 flies as negative controls. In the three cases, immunostaining was detected in the cortex (Figure 9). Aβ42 showed a highly discrete and specific labeling that was consistent with intracellular accumulation, as described [29]. ABri and ADan distribution was much more widespread, compatible with intra and extracellular accumulation. These features may explain the apparent contradiction between the extent of tissue loss and the degree of neurotoxicity among the peptides. ABri and ADan soluble species, with a more diffused distribution than Aβ42, may induce an earlier synaptic dysfunction (reflected in behavioral defects) in contrast to localized neuronal death. Moreover, brain paraffin sections showing a negative staining with ThS support the non-fibrillar form of deposition of the three amyloid peptides (Additional file 7).

## Conclusions

Our results describe a novel *Drosophila* model of mutant peptides associated with human dementias, in which the major neuropathological hallmarks are amyloid deposits and neurofibrillary tangles. In the case of ADan, the fly model may contribute to recently reported studies in transgenic mice by increasing our understanding of the molecular mechanism associated with ADan toxicity and the pathogenesis of FDD. Regarding ABri, this is the first animal model in which neurotoxicity is directly tested *in vivo*.

Although low levels of expression of the transgenes may be a drawback for an easy detection of amyloid peptide accumulation, they allow a more reliable measure of toxicity without artifacts associated with acute and high over-expression. Importantly, despite the relatively low levels of expression of our transgenes, we were able to detect toxicity in the eye and in the CNS of flies. The phenotypes, as expected, were mild as compared to published data on wild-type Aβ and Aβ mutants, and yet, sufficient to detect important similarities and differences regarding the degree of toxicity and accumulation among Aβ42, ABri and ADan. Both in the CNS and in the eye, ADan was more toxic than Aβ42 and ABri, possibly due to a faster rate of aggregation. In the CNS, ABri appeared to be more toxic than Aβ42, but the relationship between peptide levels was not straightforward, suggesting additional factors involved such as conformation or differential neuronal vulnerability. The solubility, the pattern of deposition, and the fact that all deposits in the brain and the eye were not stained by ThS, strongly suggest that toxicity of the three amyloid peptides was likely related to the accretion of soluble species. Our data on the *Drosophila* models correlate well with recent work on a double transgenic mouse model for FDD, in which enhanced tauopathy, tau truncation, and synaptic loss occurs prior to any detectable ADan deposition [15].

In summary, the *Drosophila* lines presented in this paper support the neurotoxicity of ABri and confirm the toxicity of ADan and Aβ42, suggesting that this effect may be important in the pathogenesis of human dementia associated with the slow and relentless accumulation of amyloid peptides.

## Methods

### Generation of transgenic lines and stocks

The Bri_2_-23, ABri and ADan cDNAs were obtained by PCR from BRI_2_ mutant templates [14,48]. Aβ42 cDNAs was amplified from human AβPP-CT99 fragment cloned in pGEX. The cDNA of necrotic protein signal peptide MASKVSILLLLTVHLLAAQTFAQ was obtained from the pOT Nec (GH10112). The primers’ sequences and overall strategy to generate fusion constructs are described in Additional file 1. Constructs were subcloned in the pUAST- attB expression vector. Transgenic flies were obtained by injection of embryos from line ΦX-86Fb (y w M {eGFP.vas-int. Dm} ZH-2A; +; M {RFP. attP} ZH-86Fb; +) with constructs cloned in pUAST- attB, and therefore integration took place at a specific site on chromosome 3 [42]. The *GMR*-*GAL4* and *elav*^*c155*^-*GAL4* lines were obtained from Bloomington Stock Center. The Hj2.12 line was kindly provided by Dr. Mary Konsolaki. This line has several *UAS*-Aβ42 randomly inserted on chromosome 2 (personal communication). All crosses and maintenance of flies were done at 25°C or 28°C as indicated.

### Peptides and antibodies

Synthetic Aβ1-42 was obtained from American Peptide Co. Synthetic ABri was a generous gift of Prof. Jorge Ghiso, New York University. Monoclonal anti-Aβ 6E10 was from Signet. Monoclonal antibodies anti-His were obtained from Abcam, Cambridge, MA and Qiagen, Valencia, CA. Polyclonal antisera anti-ADan and anti-ABri were generated as reported before [14].

### DNA extraction and PCR

Thirty flies were homogenized in 400 μl of Buffer A (100 mM Tris–HCl, pH 7.5, 100 mM EDTA,100 mM NaCl, 0.5% SDS) and incubated at 65°C for 30 min. Eight hundred μl of a 5 M KOAc: 6 M LiCl (1:2.5) solution were added and left 10 min on ice. After centrifugation at 14,000 rpm for 15 min, 700 μl of isopropanol were added per ml of supernatant. Samples were centrifuged at 14,000 rpm for 15 min, the pellet washed with cold ethanol and resuspended in water. PCR reactions were done using KAPA Taq (Kapa Biosystems, Woburn, MA) and run on a Bio-Rad cycler (Bio-Rad, Hercules, CA). The following primers, which are described in Additional file 1, were used: forward, Br_F1b and reverse Br_R2 for BRI_2_-23, Br_R3 for ABri, Br_R5 for ADan and R-Aβ42 for Aβ42.

### RNA extraction and quantitative real time PCR analysis

For each experiment, 30–40 flies carrying two copies of each transgene were collected and frozen. Heads were mechanically isolated and total RNA extracted using Trizol (Invitrogen Carlsbad, USA) according to the manufacturer’s protocol. An additional centrifugation step at 11,000 × g for 10 min was used to remove the cuticles prior to the addition of chloroform. The concentration of total RNA purified from each sample was measured with a Nanodrop 2000 spectrophotometer (Thermo Scientific Walthman, MA). One to 2.5 μg of total RNA were digested with DNAse I (Promega Madison, WI) immediately followed by reverse transcription using the Superscript II system (Invitrogen, Carlsbad, USA) with oligo (dT) primers. PCR reactions were done using KAPA SYBR* FAST qPCR Master Mix (2×) from KAPA BIOSYSTEMS (Woburn, MA) following the manufacturers’ instructions. Reactions were run in an Mx3005P Cycler (Stratagene, Santa Clara, CA). Data were analyzed using MxPRO-Mx3005P software. The primers used for *Nec*-Bri_2_-23, *Nec*-ABri and *Nec*-ADan transgenes were directed to: 1) the *Nec* sequence (forward: 5′-ATA CGA ATT CAT GGC GAG CAA AG-3′); and 2) a common sequence for the three peptides, C-terminal of Bri_2_-23 (reverse 5′-GTT TCCACG GCA AAT TTG TT-3′). For tubulin cDNA amplification, the following primers were used: forward 5′-GCC TGA ACA TAG CGG TGA AC-3′ and reverse 5′-ATC CCC AAC AAC GTG AAG AC-3′.

### Tissue processing, SDS-PAGE and Western blots

Fly heads were homogenized in RIPA buffer (50 mM Tris–HCl, pH 8.0, 150 mM NaCl, containing 0.5% sodium deoxycholate, 1% Triton X-100), 1% SDS, 5 mM EDTA, 5 mM EGTA, 5 μg/ml leupeptin, 10 μg/ml aprotinin, 1 μg/ml pepstatin, 50 mM sodium fluoride and 5 mM sodium orthovanadate. After sonication, homogenates were centrifuged at 14,000 rpm for 1 h. RIPA insoluble pellets were homogenized in 90% formic acid (FA) followed by centrifugation as described above. FA was evaporated by Speed Vac (Savant, SC100) and RIPA and FA-soluble proteins were re-suspended in sample buffer containing 0.1 M dithiothreitol. Protein extracts were separated on 12.5 or 15% Tris-Tricine gels and transferred onto PVDF membranes (GE Bioscience, Piscataway, NJ). Membranes were incubated with 6E10 monoclonal antibody (1/1000), anti-ABri (1/500) anti-ADan (1/1000) or a mixture of both monoclonal anti-His (1/500 each Qiagen and Abcam). Anti-actin (Sigma) was used for protein loading normalization. Immunoreactivity was detected with anti-rabbit or anti-mouse horseradish peroxidase-labeled IgG (Dako Denmark) and enhanced chemiluminescence ECL Plus (GE Bioscience Piscataway, NJ). Immunoblots were scanned with Storm 840 and band intensities quantified with ImageQuant 5.1 software (GE Bioscience Piscataway, NJ).

### Eye toxicity

Flies expressing one or two copies of the peptides in the eye (*GMR-GAL4*) were raised at 25°C or 28°C and aged for 10 days before examination under light microscope. At least 100 eyes per genotype were analyzed from 5 independent biological experiments. Due to the high penetrance of phenotypes (90-100%), 8–10 eyes per genotype were randomly selected for Environmental Scanning Electron Microscopy (ESEM) analysis. Adult flies were immobilized on the ESEM mount using water-based colloidal carbon glue for proper orientation. The electro-scan was performed with an ESEM microscope model XL30 (Philips) at 20.0 kV and 0.9 Torr in the auxiliary mode. This technology did not require metal coating of the specimen. To analyze toxicity in a quantitative manner, eyes were properly oriented and a comparable area for each genotype was selected. The number of ommatidia, fused ommatidia and bristles were also counted in the selected areas. Eight fly eyes were analyzed per genotype. All the counting and measurements were done as blind experiments.

### Histology and immunostaining

Fly heads were fixed in Carnoy solution (60% ethanol, 30% chloroform, 10% acetic acid) at 4°C overnight and dehydrated in ethanol. Samples were treated for 30 min with butanol:ethanol (1:1), butanol:toluene (1:1), toluene, and finally soaked in toluene: paraffin (1:1) for 30 min at 65°C. After incubation for 2 h in pure paraffin, heads were embedded and cut in 7-μm serial frontal sections. After H&E staining, images were captured using an OLYMPUS B × 50 Microscope and analyzed with the Image Pro Plus software (Media Cybernetics). Neurodegeneration was quantified and reported as the percentage of area lost in the tissue. The ratio was calculated by dividing the area of vacuolization (each vacuole with a diameter of at least 3 μm) by the total area of the brain section. At least 8 brains per genotype were analyzed. ThS staining was used for the detection of amyloid fibrillar deposits. The tissue was deparaffinized and incubated in 50% ethanol containing 1% ThS (Sigma St. Louis, MO) for 20 min. After washing in 50% ethanol and PBS, tissue sections were analyzed using a confocal Zeiss LSM510 microscope. Immunostaining was performed by incubating sections with anti-Aβ monoclonal 6E10 (1/500) and protein A-sepharose affinity-purified anti-ADan and anti-ABri antibodies (1/100). Immunoreactivity was detected with biotinylated anti-mouse or anti-rabbit IgG followed by incubation with avidin–biotin complex (Vector Laboratories, Burlingame, CA). The reaction products were visualized with 0.05% diaminobenzidine tetrahydrochloride, 0.01% hydrogen peroxide and, in some cases with nickel enhancement. Negative controls included incubation with primary unrelated antibodies and immunostainig of *GAL4*/Bri_2_-23 fly sections with amyloid peptides-specific antibodies.

### Climbing assay (rapid iterative negative geotaxis, RING)

Flies expressing one or two copies of Bri_2_-23, Aβ42, ABri and ADan peptides in the CNS (with *elav-GAL4*) were raised at 25°C and incubated at 28°C in groups of 40 males in 4-inch glass vials with food replacement every 3–4 days. Vertical mobility was tested using the RING assay as described [49]. Briefly, the day before the assay 10 males per genotype were randomly selected under CO_2_. The following day, each group was transferred into empty vials without anesthesia and the vials were loaded into the RING apparatus. The apparatus was tapped three times in rapid succession to initiate a negative geotaxis response. After 4 sec, digital images were taken. The climbed distance in cm was measured for each fly and the average height per genotype calculated using the Scion Image software. Data from 5 technical replicates from each of 3 independent biological experiments per genotype were analyzed.

### Phototaxis assay

Flies expressing one copy of Bri_2_-23, Aβ42, ABri and ADan in the CNS (with elav-GAL4) were raised and kept at 25°C. Phototaxis was performed as described [44]. Briefly, the day before the assay, 30 males per genotype were randomly selected under CO_2_. The following day, 15 min before testing, flies were transferred to darkness. Further manipulations were performed under red light. For the assay, flies were transferred to empty tubes and for 2 min they were able to move towards a collecting tube which had a white cold light at the end of it. The number of flies that moved to the collecting tube, towards the light (defined as a positive response) was counted and the results were expressed as the percentage of flies that responded to light of the total number of animals per assay. Data from 2 technical replicates from each of 3 independent biological experiments per genotype were analyzed.

### Circadian locomotor activity

Fly activity was monitored as described [44]. Briefly, newly eclosed male flies were trained under 12 h light-darkness (LD) cycles until the beginning of the experiment. Then, young (5 days-old) and aged (21 days-old) flies were placed in glass tubes and monitored for activity with infrared detectors. Fly activity was monitored under LD conditions for 3 days and then released into constant darkness (DD) for at least one week employing commercially available activity monitors (TriKinetics, Waltham, MA). Period and rhythmicity were estimated using the ClockLab software (Actimetrics, Evanston, IL) from data collected under DD. Each experiment was repeated at least two times from two independent biological replicates.

### ELISA

Five fly heads from our line *GMR*/Aβ42 carrying two copies of the transgene and 5 heads from the *GMR/*Hj2.12 line were homogenized and proteins extracted in 50 mM Tris–HCl, pH 8 containing 5 M guanidine and a cocktail of proteases inhibitors (Sigma St. Louis, MO). After centrifugation at 10,000 × g, proteins in the supernatant were quantified using a bicinchoninic acid kit (Thermo Scientific, Rockford, IL). Aβ42 concentration was determined using a commercial capture ELISA kit (Invitrogen Frederick, MD) following the manufacturer’s instructions.

### Statistical analysis

Data were analyzed by Student’s t-test, One-way ANOVA, and Two-way repeated measures ANOVA, followed by post-hoc tests. Graph Pad Prism v.5 software was used. Statistical results were presented as means ± SEM. Asterisks indicate levels of significance (*p <0.05, **p <0.01 and ***p < 0.001).

## Abbreviations

Aβ: Amyloid β; ABri: Amyloid Bri; ADan: Amyloid Dan; AD: Alzheimer’s disease; ADAM10: A disintegrin and metalloproteinase domain-containing protein 10; AβPP: Amyloid β-precursor protein; DD: Constant darkness; ELISA: Enzyme-linked immuno-sorbent assay; ESEM: Environmental scanning electron microscopy; FBD: Familial British dementia; FDD: Familial Danish dementia; GMR: Glass-multiple-reporter; H&E: Hematoxylin-eosin; ITM2B: Integral trans-membrane protein 2B; LD: Light-darkness; Nec: Necrotic; Bri2-23: The BRI_2_ wild-type derived peptide of 23 residues; PBS: Phosphate buffered saline; PBS-T: PBS containing 1% Triton X-100; PC: Pro-protein convertases; PCR: Polymerase chain reaction; pdf: Pigment-dispersing factor; p. e: Post-eclosion; PVDF: Polyvinylidene fluoride; QRT: Quantitative real time; RING: Rapid iterative negative geotaxis; SDS: Sodium dodecyl sulfate; sp: Synthetic peptide; ThS: Thioflavine S; UAS: Upstream-activator sequence.

## Competing interests

The authors have no competing interests.

## Authors’ contributions

MSM analyzed toxicity, performed histological analysis, Western blots and wrote the paper; AFG performed immunohistochemistry and Western blots; LAA generated and maintained transgenic lines, CR and OLP performed tissue sections and histological data analysis; LM, RV, MFC were involved in the experimental design; EMC designed experiments and wrote the paper. All authors read and approved the final manuscript.

## Supplementary Material

Additional file 1 **Primers used for constructs generation.** Table with the sequences of all primers used in this study and PCR strategy. Click here for file

Additional file 2 **Western blots of amyloid peptides in SDS-insoluble factions from *Drosophila *** **eyes.** Accumulation of Aβ42 and ADan in the formic acid-soluble fraction. Non-specific immunoreactive bands in Western blots from RIPA-soluble homogenates. Click here for file

Additional file 3 **Toxicity in the *Drosophila *** **eye.** Toxicity of *GMR-*GAL/+ as compared to *GMR*-*GAL4*/Bri_2_-23 lines at 28°C for 10 days; dose dependent Aβ42 toxicity in the eye and number of ommatidia in transgenic vs non-transgenic lines. Click here for file

Additional file 4 **Accumulation of untagged and His-tagged amyloid peptides.** Western blot anti specific peptides from fly heads expressing tag and untagged peptides. Click here for file

Additional file 5 **Geotaxis and Phototaxis statistical analysis.** Two-way repeated measures ANOVA, followed by comparison tests for all genotypes and time points. Click here for file

Additional file 6 ***pdf*-neurons are resistant to amyloid peptides toxicity.** Figure showing the circadian locomotor activity of transgenic flies. Click here for file

Additional file 7 **Thioflavine S-negative accumulation of Aβ, ABri and ADan in the CNS of *Drosophila.*** Figure showing negative Thioflavine-S staining of brain sections from *Drosophila* transgenic lines. Click here for file
