# Supplementary material for: Amyloid peptides ABri and ADan show differential neurotoxicity in transgenic Drosophila models of familial British and Danish dementia
Source: Mol Neurodegener. 2014 Jan 9;9:5. doi: 10.1186/1750-1326-9-5 (PMC3898387; doi:10.1186/1750-1326-9-5)
Supplement: Additional file 1 — Primers used for constructs generation. Table with the sequences of all primers used in this study and PCR strategy. [file 1750-1326-9-5-S1.pdf]

Additional file 1:

Primers used for constructs generation

Template	Primers Sequence	Product
pOT Nec (GH10112)	Forw:**Br_F1b**: 5'-ATA CGA ATT CAT GGC GAG CAA AG-3'<br>Rev: **Br_R4**: 5'-CAA TTG CTG GCT TCC TGG GCG AAG GTC TG-3.<br>His Rev: **Br_R1**: 5'-TCG TGG TGG TGG TGA TGA TGC TGG GCG AAG GTC-3'.	**NEC**<br>**NEC-HIS**
BRI2 mutant templates	Forw: **Br_F3**: 5'-CAG ACC TTC GCC CAG GAA GCC AGC AAT TG-3'.<br>His Forw: **Br_F2**: 5'-CAT CAT CAC CAC CAC CAC GAA GCC AGC AAT TGT-3'.<br>Rev: **Br_R2**: 5'-GAT TCT CGA GTC AAG AAC AAA TTA AAG-3'.	**BRI_2 -23**<br>**HIS- BRI_2 -23**
BRI2 mutant templates	Forw: **Br_F3**: 5'-CAG ACC TTC GCC CAG GAA GCC AGC AAT TG-3'.<br>His Forw**: Br_F2**: 5'-CAT CAT CAC CAC CAC CAC GAA GCC AGC AAT TGT-3'.<br>Rev: **Br_R3**: 5'-GAT TCT CGA GTT AAT TTT CCT CAA TAA TG-3'	**ABri**<br>**HIS-ABri**
BRI2 mutant templates	Forw: **Br_F3**: 5'-CAG ACC TTC GCC CAG GAA GCC AGC AAT TG-3'.<br>His Forw: **Br_F2**: 5'-CAT CAT CAC CAC CAC CAC GAA GCC AGC AAT TGT-3'.<br>Rev: **Br_R5**: GAT TCT CGA GTC AAT AAT GTT TTT CTT GAC TGT-3'	**ADan**<br>**HIS-ADan**
pGEX APP-CT99	Forw:**F-Aβ42**: 5´-CAG-ACC-TTC-GCC-CAG-GAT-GCA-G -3´<br>His Forw: **F-HIS Aβ42**: 5´-CAT-CAT-CAC-CAC-CAC-CAC-GAT-G -3´<br>Rev: **R-Aβ42**: 5´- GAT TCT CGA GTC ACG CTA TGA CAA CAC-3´	**Aβ42**<br>**HIS-Aβ42**

The signal peptide cDNA from the *Drosophila* necrotic protein (Nec) was obtained from the pOT Nec (GH10112) by PCR using the following primers: forward Br_F1b and reverse: Br_R4. To generate the NEC-His construct we used forward Br_F1b and reverse: Br_R1.

The BRI_2-23, ABri and ADan cDNAs were obtained by PCR from BRI2 templates using the following primers: for BRI_2-23, ABri and ADan, forward: Br_F3 and reverse Br_R2, Br_R3 and Br_R5 respectively. (see Table) For His-tag constructs, forward, Br_F2 and reverse Br_R2, Br_R3 and Br_R5 respectively. The Aβ42 cDNAs was amplified from human APP-CT99 fragment cloned in pGEX using primer forward F_Aβ42 and reverse R_Aβ42, and for the His-tag version forward: F_HIS-Aβ42 and reverse R_Aβ42.

To get the Nec-peptide or Nec- His-peptide construct a nested PCR was performed mixing the PCR products from the previous reactions:

1st PCR products	Primers used	Final Construct
NEC + BRI_2-23	Forw: **Br_F1b** and rev: **Br_R2**	NEC- BRI_2-23
NEC-HIS + HIS- BRI_2-23	Forw: **Br_F1b** and rev: **Br_R2**	NEC-HIS- BRI_2-23
NEC + ABri	Forw: **Br_F1b** and rev **Br_R3**	NEC-ABri
NEC-HIS +HIS- ABri	Forw: **Br_F1b** and rev**:Br_R3**	NEC-HIS- ABri
NEC + ADan	Forw: **Br_F1b** and rev**:Br_R5**	NEC-ADan
NEC-HIS + HIS-ADan	Forw: **Br_F1b** and rev**:Br_R5**	NEC-HIS-ADan
NEC + Aβ42.	Forw: **Br_F1b** and rev:**R-Aβ42**	NEC-Aβ42.
NEC-HIS + HIS-Aβ42.	Forw: **Br_F1b** and rev:**R-Aβ42**	NEC-HIS-Aβ42.

This second PCR product was cloned in the pUAST-attb.
