# Supplementary material for: Amyloid peptides ABri and ADan show differential neurotoxicity in transgenic Drosophila models of familial British and Danish dementia
Source: Mol Neurodegener. 2014 Jan 9;9:5. doi: 10.1186/1750-1326-9-5 (PMC3898387; doi:10.1186/1750-1326-9-5)
Supplement: Additional file 2 — Western blots of amyloid peptides in SDS-insoluble factions from Drosophila eyes. Accumulation of Aβ42 and ADan in the formic acid-soluble fraction. Non-specific immunoreactive bands in Western blots from RIPA-soluble homogenates. [file 1750-1326-9-5-S2.pdf]

Additional file 2

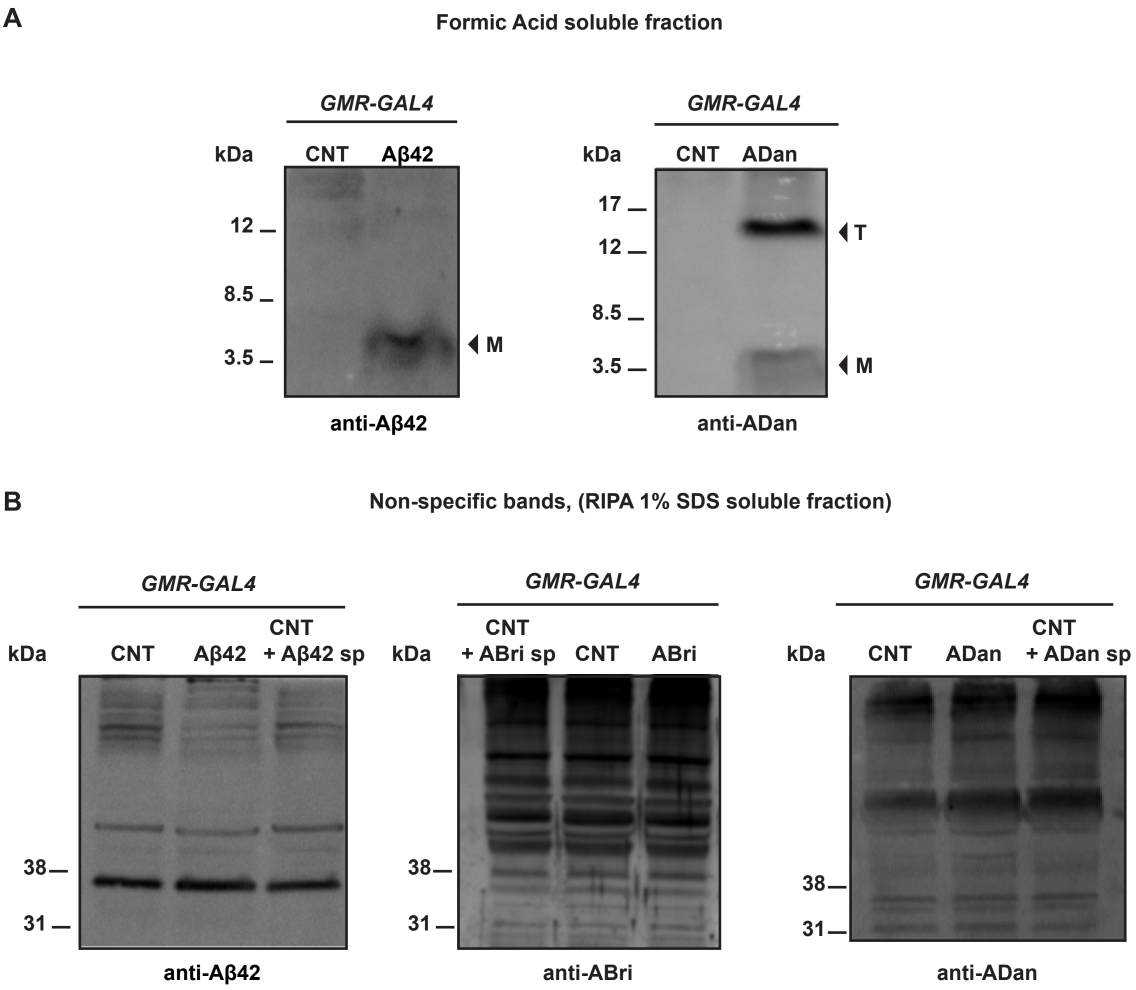

Supp. Figure 1. Western blots showing RIPA-insoluble amyloid peptides after solubilization in formic acid. **A**, Aβ42 monomers were detected using 6E10 antibody (arrowhead). Monomers (M) and putative tetramers (T) were detected using anti ADan antibody. Note that FA soluble proteins from 200 heads homogenates were loaded, as compared to 15 heads used for the RIPA-soluble proteins as shown in Figure 3. **B**, Upper part of the Western blots shown in Figure 3, in which non-specific inmunoreactivity bands are similar in all genotypes. Note the absence of specific bands suggestive of high-order oligomers for the three amyloid peptides.
