# Supplementary material for: Amyloid peptides ABri and ADan show differential neurotoxicity in transgenic Drosophila models of familial British and Danish dementia
Source: Mol Neurodegener. 2014 Jan 9;9:5. doi: 10.1186/1750-1326-9-5 (PMC3898387; doi:10.1186/1750-1326-9-5)
Supplement: Additional file 3 — Toxicity in the Drosophila eye. Toxicity of GMR-GAL/+ as compared to GMR-GAL4/Bri2-23 lines at 28°C for 10 days; dose dependent Aβ42 toxicity in the eye and number of ommatidia in transgenic vs non-transgenic lines. [file 1750-1326-9-5-S3.pdf]

Additional file 3

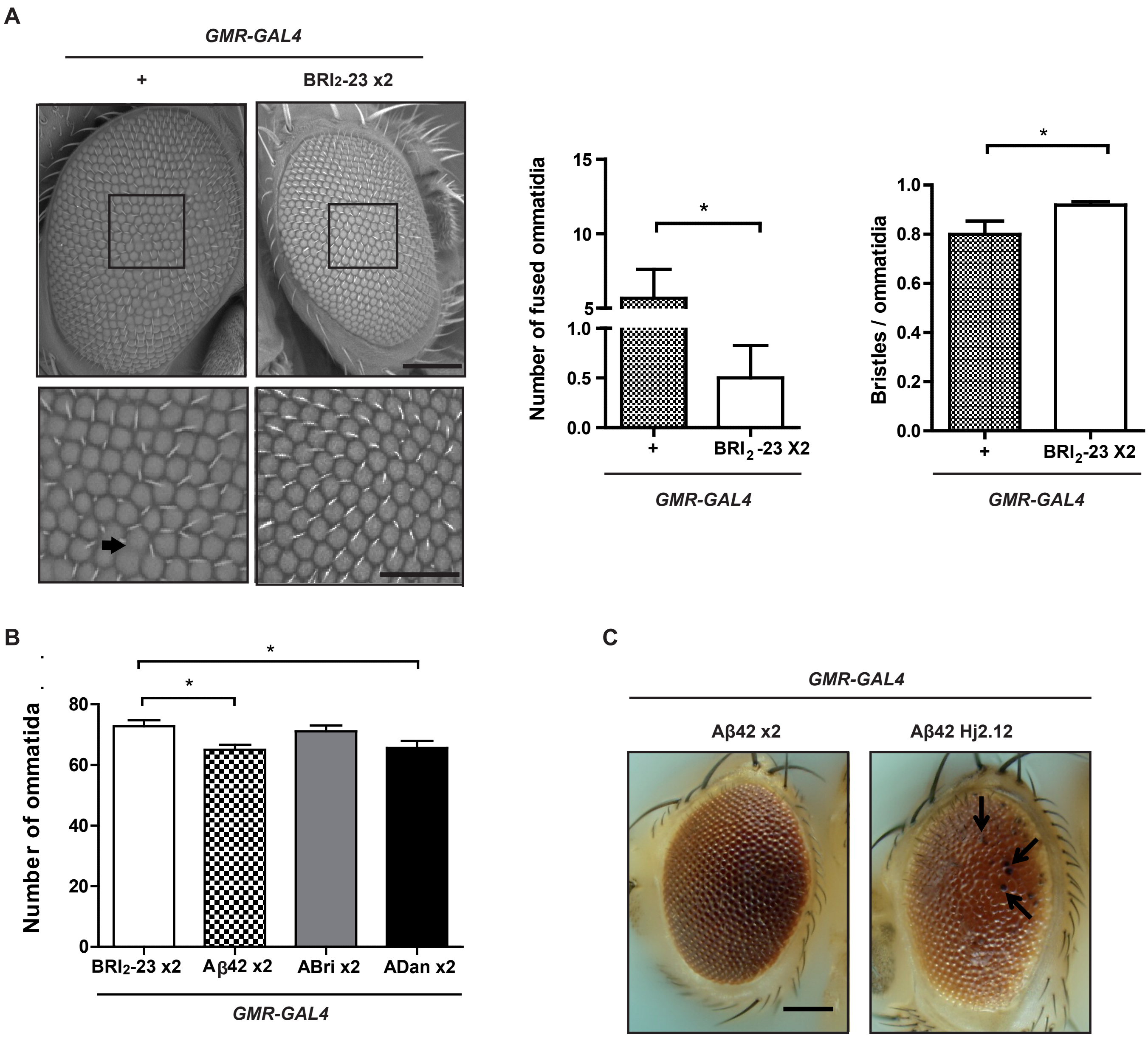

Supp. Figure 2. Toxicity in the Drosophila eye. A, Left panel, ESEM showing *GMR*-GAL4/+ as compared to *GMR-GAL4*/Bri$_2$-23 lines and right panel, quantification of fused ommatidia and number of bristles per ommatidia. Bars represent the mean ± SEM. Asterisks indicate a statistically significant difference (*$p<0.05$, Student's t-test). **B**, Number of ommatidia in a central selected area (135 x135 µm) in the eyes of transgenic and non-transgenic lines. Bars represent the mean ± SEM. Asterisks indicate a statistically significant difference (*$p<0.05$, One-way ANOVA, followed by Dunnett`s comparison). **C,** Aβ42 toxicity in the eye. At 25°C, *GMR-GAL4*/Aβ42 Hj2.12 developed a rough eye with areas of necrosis (arrows) as compared to a normal phenotype in our *GMR-GAL4*/Aβ42 x2 line. In Aβ42 Hj2.12 Aβ42 levels are 2 -fold higher as compared to our *GMR-GAL4*/Aβ42 x2 line**.** Scale bar =100 µm.
