# Supplementary material for: Amyloid peptides ABri and ADan show differential neurotoxicity in transgenic Drosophila models of familial British and Danish dementia
Source: Mol Neurodegener. 2014 Jan 9;9:5. doi: 10.1186/1750-1326-9-5 (PMC3898387; doi:10.1186/1750-1326-9-5)
Supplement: Additional file 4 — Accumulation of untagged and His-tagged amyloid peptides. Western blot anti specific peptides from fly heads expressing tag and untagged peptides. [file 1750-1326-9-5-S4.pdf]

**Additional file 4**

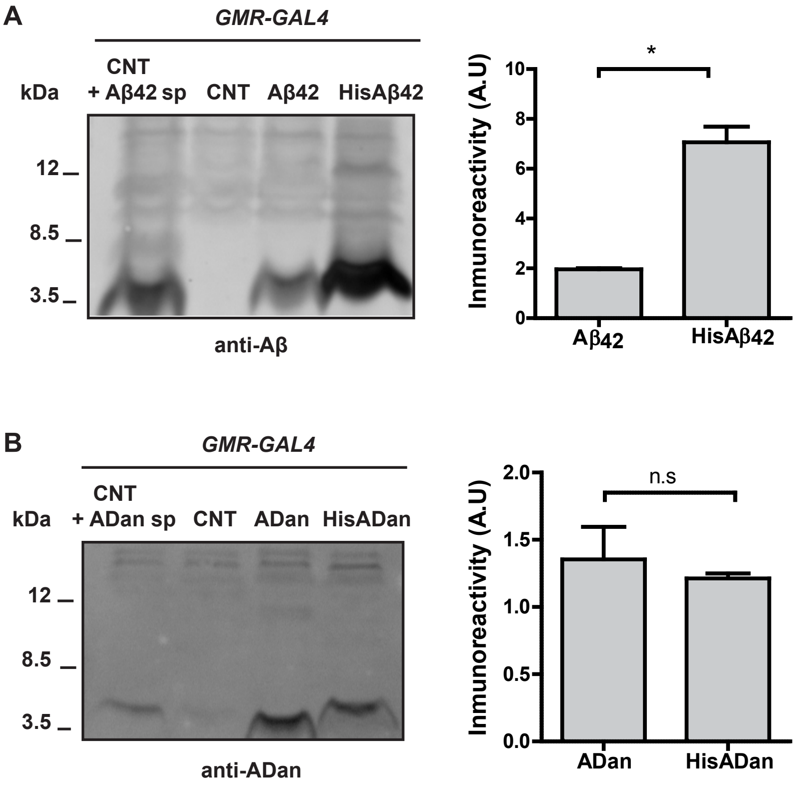


**Supp. Figure 3. Accumulation of untagged and His-tagged amyloid peptides. A**, Western blot with anti-Aβ from fly heads expressing two copies of Aβ42 or HisAβ42 as compared to non-transgenic flies. **B**, Western blot with anti- ADan from fly heads expressing two copies of ADan or HisADan compared to non-transgenic flies. On the right of each panel, quantification of immunoreactive bands. Bars represent the mean ± SEM of three experiments. Asterisk indicates a statistically significant difference (*$p<0.05$, Student's t-test)
