# Supplementary material for: Amyloid peptides ABri and ADan show differential neurotoxicity in transgenic Drosophila models of familial British and Danish dementia
Source: Mol Neurodegener. 2014 Jan 9;9:5. doi: 10.1186/1750-1326-9-5 (PMC3898387; doi:10.1186/1750-1326-9-5)
Supplement: Additional file 5 — Geotaxis and Phototaxis statistical analysis. Two-way repeated measures ANOVA, followed by comparison tests for all genotypes and time points. [file 1750-1326-9-5-S5.pdf]

Additional file 5

Statistical analysis of geotaxis assay.

	***elav-GAL4*/BRI$_2$-23 x2**			***elav-GAL4*/Aβ42 x2**			***elav-GAL4*/ABri x2**			***elav-GAL4-*/ADan x2**		
**Age**	**Mean**	**SEM**	**N**	**Mean**	**SEM**	**N**	**Mean**	**SEM**	**N**	**Mean**	**SEM**	**N**
7	4,656415	0,2192307	3	4,616077	0,1463194	3	3,859194	0,1895314	3	2,519642	0,7548203	3
15	3,834954	0,1079299	3	3,588669	0,160441	3	2,380756	0,2895665	3	1,418514	0,3269992	3
21	2,991749	0,1855949	3	1,468902	0,2733525	3	1,13085	0,3163382	3	1,393163	0,1397144	3

Two-way, repeated measures, ANOVA, followed by Bonferroni `s Multiple Comparisons, (GraphPad, Prism 5)

elav-BRI2-23 vs elav-Aβ42				
genotype	elav-BRI2-23	elav-Aβ42	Difference	95% CI of diff,
7	4,656	4,616	-0,04034	-1,488 to 1,407
15	3,835	3,589	-0,2463	-1,694 to 1,202
21	2,992	1,469	-1,523	-2,971 to -0,07504
genotype	Difference	t	P value	Summary
7	-0,04034	0,09286	P > 0,05	ns
15	-0,2463	0,5670	P > 0,05	ns
21	-1,523	3,506	P<0,01	**
elav-BRI2-23 vs elav-ABri				
genotype	elav-BRI2-23	elav-ABri	Difference	95% CI of diff,
7	4,656	3,859	-0,7972	-2,245 to 0,6506
15	3,835	2,381	-1,454	-2,902 to -0,006394
21	2,992	1,131	-1,861	-3,309 to -0,4131
genotype	Difference	t	P value	Summary
7	-0,7972	1,835	P > 0,05	ns
15	-1,454	3,348	P<0,01	**
21	-1,861	4,284	P<0,001	***
elav-BRI2-23 vs elav-ADan				
genotype	elav-BRI2-23	elav-ADan	Difference	95% CI of diff,
7	4,656	2,520	-2,137	-3,585 to -0,6890
15	3,835	1,419	-2,416	-3,864 to -0,9686
21	2,992	1,393	-1,599	-3,046 to -0,1508
genotype	Difference	t	P value	Summary
7	-2,137	4,919	P<0,001	***
15	-2,416	5,563	P<0,001	***
21	-1,599	3,680	P<0,01	**
elav-Aβ42 vs elav-ABri				
genotype	elav-Aβ42	elav-ABri	Difference	95% CI of diff,

7	4,616	3,859	-0,7569	-2,205 to 0,6909
15	3,589	2,381	-1,208	-2,656 to 0,2399
21	1,469	1,131	-0,3381	-1,786 to 1,110
genotype	Difference	t	P value	Summary
7	-0,7569	1,742	P > 0,05	ns
15	-1,208	2,781	P < 0,05	*
21	-0,3381	0,7783	P > 0,05	ns
elav-Aβ42 vs elav-ADan				
genotype	elav-Aβ42	elav-ADan	Difference	95% CI of diff,
7	4,616	2,520	-2,096	-3,544 to -0,6486
15	3,589	1,419	-2,170	-3,618 to -0,7224
21	1,469	1,393	-0,07574	-1,524 to 1,372
genotype	Difference	t	P value	Summary
7	-2,096	4,826	P<0,001	***
15	-2,170	4,996	P<0,001	***
21	-0,07574	0,1744	P > 0,05	ns
elav-ABri vs elav-ADan				
genotype	elav-ABri	elav-ADan	Difference	95% CI of diff,
7	3,859	2,520	-1,340	-2,787 to 0,1083
15	2,381	1,419	-0,9622	-2,410 to 0,4856
21	1,131	1,393	0,2623	-1,185 to 1,710
genotype	Difference	t	P value	Summary
7	-1,340	3,084	P < 0,05	*
15	-0,9622	2,215	P > 0,05	ns
21	0,2623	0,6039	P > 0,05	ns

Statistical analysis of Phototaxis assay.

	***elav-GAL4*/BRI_2-23 x1**			***elav-GAL4*/Aβ42 x1**			***elav-GAL4*/ABri x1**			***elav-GAL4-*/ADan x1**		
**Age**	**Mean**	**SEM**	**N**	**Mean**	**SEM**	**N**	**Mean**	**SEM**	**N**	**Mean**	**SEM**	**N**
5	68,30296	3,884292	3	77,92404	7,68356	3	73,13538	5,379315	3	52,28471	14,59665	3
25	71,93841	5,813278	3	68,99335	3,707666	3	65,77213	4,37508	3	46,19212	6,752286	3

Two-way, repeated measures, ANOVA, followed by Bonferroni `s Multiple Comparisons, (GraphPad, Prism 5)

elavGAL4-BRI_2-23 vs elavGAL4-Aβ42				
Column Factor	elavGAL4-BRI_2-23	elavGAL4-Aβ42	Difference	95% CI of diff,
5	68,30	77,92	9,621	-24,93 to 44,17
25	71,94	68,99	-2,945	-37,50 to 31,61
Column Factor	Difference	t	P value	Summary
5	9,621	0,9297	P > 0,05	ns

25	-2,945	0,2846	P > 0,05	ns
elavGAL4-BRI$_2$-23 vs elavGAL4-ABri				
Column Factor	elavGAL4-BRI$_2$-23	elavGAL4-ABri	Difference	95% CI of diff,
5	68,30	73,14	4,832	-29,72 to 39,38
25	71,94	65,77	-6,166	-40,72 to 28,38
Column Factor	Difference	t	P value	Summary
5	4,832	0,4669	P > 0,05	ns
25	-6,166	0,5958	P > 0,05	ns
elavGAL4-BRI$_2$-23 vs elavGAL4-ADan				
Column Factor	elavGAL4-BRI$_2$-23	elavGAL4-ADan	Difference	95% CI of diff,
5	68,30	52,28	-16,02	-50,57 to 18,53
25	71,94	46,19	-25,75	-60,30 to 8,804
Column Factor	Difference	t	P value	Summary
5	-16,02	1,548	P > 0,05	ns
25	-25,75	2,488	P < 0,05	*
elavGAL4-Aβ42 vs elavGAL4-ABri				
Column Factor	elavGAL4-Aβ42	elavGAL4-ABri	Difference	95% CI of diff,
5	77,92	73,14	-4,789	-39,34 to 29,76
25	68,99	65,77	-3,221	-37,77 to 31,33
Column Factor	Difference	t	P value	Summary
5	-4,789	0,4627	P > 0,05	ns
25	-3,221	0,3113	P > 0,05	ns
elavGAL4-Aβ42 vs elavGAL4-ADan				
Column Factor	elavGAL4-Aβ42	elavGAL4-ADan	Difference	95% CI of diff,
5	77,92	52,28	-25,64	-60,19 to 8,911
25	68,99	46,19	-22,80	-57,35 to 11,75
Column Factor	Difference	t	P value	Summary
5	-25,64	2,477	P < 0,05	*
25	-22,80	2,203	P > 0,05	ns
elavGAL4-ABri vs elavGAL4-ADan				
Column Factor	elavGAL4-ABri	elavGAL4-ADan	Difference	95% CI of diff,
5	73,14	52,28	-20,85	-55,40 to 13,70
25	65,77	46,19	-19,58	-54,13 to 14,97
Column Factor	Difference	t	P value	Summary
5	-20,85	2,015	P > 0,05	ns
25	-19,58	1,892	P > 0,05	ns
