# Supplementary material for: Amyloid peptides ABri and ADan show differential neurotoxicity in transgenic Drosophila models of familial British and Danish dementia
Source: Mol Neurodegener. 2014 Jan 9;9:5. doi: 10.1186/1750-1326-9-5 (PMC3898387; doi:10.1186/1750-1326-9-5)
Supplement: Additional file 6 — pdf -neurons are resistant to amyloid peptides toxicity. Figure showing the circadian locomotor activity of transgenic flies. [file 1750-1326-9-5-S6.pdf]

Additional file 6

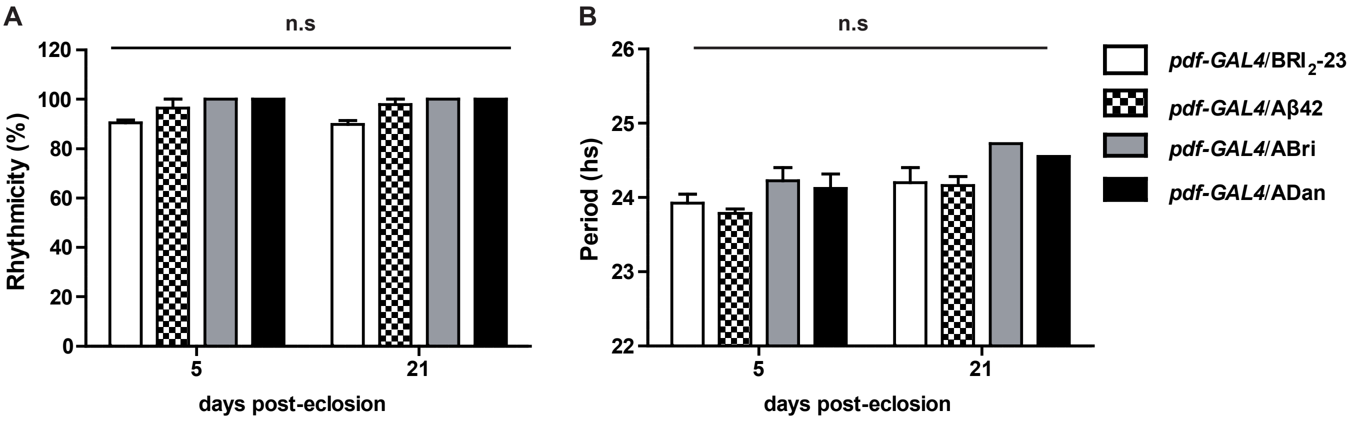

Supp. Figure 4. The over expression of two copies of Aβ42, ABri and ADan in *pdf*-neurons did not affect the circadian behavior. A, Percentage of rhythmic flies expressing 2 copies of amyloid peptides in *pdf* neurons at 5 and 21 days of age. The results show no differences among genotypes (bars represent the mean ± SEM of two independent experiments). **B**, Average periods for BRI_2-23, Aβ42, ABri and ADan-carrying lines. There is no difference in period values among the lines (bars represent the mean ± SEM of two independent experiments).
