# Supplementary material for: Amyloid peptides ABri and ADan show differential neurotoxicity in transgenic Drosophila models of familial British and Danish dementia
Source: Mol Neurodegener. 2014 Jan 9;9:5. doi: 10.1186/1750-1326-9-5 (PMC3898387; doi:10.1186/1750-1326-9-5)
Supplement: Additional file 7 — Thioflavine S-negative accumulation of Aβ, ABri and ADan in the CNS of Drosophila. Figure showing negative Thioflavine-S staining of brain sections from Drosophila transgenic lines. [file 1750-1326-9-5-S7.pdf]

Additional file 7

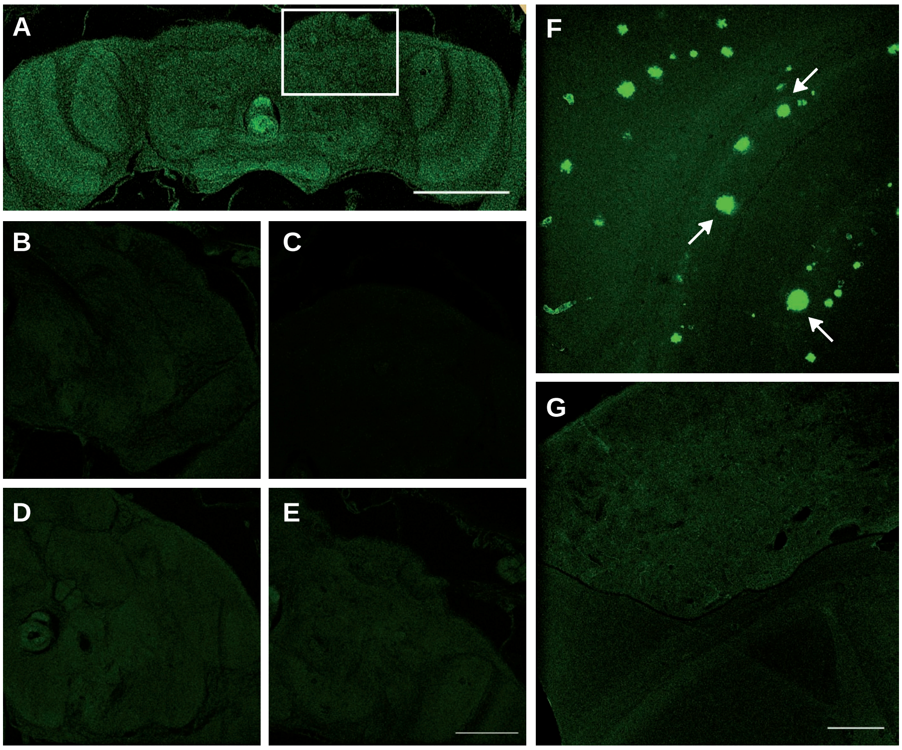

Supp. Figure 5. Thioflavine S-negative accumulation of Aβ42, ABri and ADan in the CNS of Drosophila. A, Whole brain section showing selected area (scale bar=100 μm) and **B-E** brain sections of flies expressing two copies of BRI_2-23, Aβ42, ABri and ADan respectively, did not show positive ThS staining (scale bar=50 μm). **F**, Brain section from a 27 months-old Tg2576 mouse, used as a positive control, revealed ThS-positive amyloid plaques (arrows). **G**, brain section from an age-matched non-transgenic mouse littermate as a negative control (scale bar=200 μm)
